# Prognosis in Hepatic Abscess

**Published:** 1870-07

**Authors:** 


### Prognosis in Hepatic Abscess.

Dr. W. Stewart, in the *Lancet*, states as the result of a large experience in India, that where hepatic abscesses burst through the right lung, recovery is not uncommon under ordinary restorative and expectant treatment, while in cases where the discharge took place through other channels. "as into the traverse colon, stomach, etc., or externally through the parietes of the thorax or abdomen—whether naturally or by artificial opening," he has not met with a favorable termination. He hence concludes that "prognosis is favorable in uncomplicated cases, when the abscess makes its way through the lung. In such cases the abscess occupies the upper or convex portion of the liver, near the suspensory ligament; adhesive inflammation occurs on its outer surface; the diaphragm forms a part of the sac, and its substance is gradually removed by progressive absorption. If, at the same time, adhesion takes place between the diaphragmatic and pulmonary pleura, the abscess will open into the parenchyma of the lung, and be discharged more or less completely by expectoration. In such cases, the matter may escape by a small opening directly into a bronchical tube, or filter through immeasurably small orifices into the air-cells; and the process of filtration and aspiration, if I may so express it, following on the respiratory acts, may be the reason why such abscesses do not have an unhealthy action—ordinary atmospheric air carrying 'septic germs' being excluded — the products of respiration alone taking the place of the expectorated matter, and being continually renewed by the same process."—*New York Medical Gazette.*

